# Haptoglobin phenotype prevalence and cytokine profiles during *Plasmodium falciparum* infection in Dogon and Fulani ethnic groups living in Mali

**DOI:** 10.1186/1475-2875-12-432

**Published:** 2013-11-25

**Authors:** Olaf Perdijk, Charles Arama, Pablo Giusti, Bakary Maiga, Marita Troye-Blomberg, Amagana Dolo, Ogobara Doumbo, Jan-Olov Persson, Stéphanie Boström

**Affiliations:** 1 Department of Molecular Biosciences, The Wenner-Gren Institute, Stockholm University, Stockholm, Sweden; 2 Cell Biology and Immunology group, Wageningen University, Wageningen, The Netherlands; 3 Department of Epidemiology of Parasitic Diseases, Faculty of Medicine, Pharmacology & Dentistry, Malaria Research & Training Centre, University of Bamako, Bamako, Mali; 4 Division of Mathematical Statistics, Department of Mathematics, Stockholm University, Stockholm, Sweden

**Keywords:** Fulani, Dogon, *Plasmodium falciparum*, Haptoglobin phenotypes, Cytokines, sCD163, Mali

## Abstract

**Background:**

The Fulani are known to have a lower parasitaemia and less clinical episodes of malaria as compared to the Dogon sympatric ethnic group, living in Mali. Higher circulating malaria-specific antibody titers and increased pro-inflammatory cytokine levels have been shown in Fulani individuals. Several studies have tried to link haptoglobin (Hp) phenotypes with susceptibility to malaria, but without consensus. This study investigated the role of Hp phenotypes and cytokine levels in Dogon and Fulani during asymptomatic *Plasmodium falciparum* infection.

**Methods:**

Two different cohorts were combined in this study: a 2008 cohort with 77 children aged between two and ten years and a 2001 cohort, with 82 children and adults, aged between 11 and 68 years. Hp phenotypes in plasma were measured by Western Blot. Circulating levels of sCD163, IL-6, IL-10, IFN-γ and TNF were measured by ELISA. Multiple regression analysis was performed to associate Hp phenotypes with cytokine profiles. In addition, *in vitro* stimulation of peripheral blood mononuclear cells (PBMCs) with Hp:Hb complexes was performed and cytokine release in corresponding supernatants were measured using cytometric bead array.

**Results:**

The results revealed a higher Hp2-2 phenotype prevalence in the Fulani. The Hp2-2 phenotype was associated with a higher susceptibility to *P. falciparum* infection in Dogon, but not in Fulani. In concordance with previous studies, Fulani showed increased inflammatory mediators (IL-6, IFN-γ) and additionally also increased sCD163 levels compared to Dogon, irrespective of infection. Furthermore, infected individuals showed elevated sCD163 levels compared to uninfected individuals, in both Fulani and Dogon. Multiple regression analysis revealed that the Hp1-1 phenotype was associated with higher levels of TNF and IFN-γ, as compared to the Hp2-2 phenotype. *In vitro* stimulation of PBMCs with Hb:Hp1-1 complexes resulted in a pro-inflammatory cytokine profile, whilst stimulation with Hb:Hp2-2 complexes showed a more balanced profile.

**Conclusions:**

Ethnicity might be an important confounder on the Hp phenotype-dependent susceptibility to malaria and future studies could consider taking this into account when designing new immunological studies. Although, the relatively small sample size used in this study warrens for precautions in the interpretation of the data and these findings should ideally be validated in a bigger cohort.

## Background

Malaria is known to be one of the strongest selective pressures on the human genome [1]. As a possible consequence, sympatric ethnic groups can differ in their susceptibility to malaria. The Fulani ethnic group from Mali has previously been shown to be less susceptible to the *Plasmodium falciparum* parasite as reflected by a lower parasitaemia (detected by microscopy) and less clinical episodes as compared to their neighbouring sympatric group, the Dogon [2]. The Fulani have also been shown to have higher plasma levels of anti-malaria-specific antibody titers and inflammatory cytokine levels as compared to the Dogon [3,4]. Furthermore, mononuclear cells from Fulani individuals have a ten-fold higher IFN-γ production after stimulation with late-stage infected red blood cell compared to Dogon [5]. In addition, the two groups respond differently in their antigen-presenting cell subset activation upon *P. falciparum* infection as well as in the response to certain toll-like receptor ligands [6].

*Plasmodium falciparum* has a complex life cycle and is dependent on both humans and mosquitoes as hosts for its survival. In the human body, the erythrocytic cycle takes place in which merozoites infect red blood cells that mature into schizonts. After the rupture of the schizonts, newly made merozoites are released into the bloodstream and can infect new erythrocytes, thereby completing the erythrocytic cycle of the parasite [7]. During this rupture, haemoglobin (Hb) is released into the bloodstream. The haem group is lipophilic and can disrupt lipid bilayers of cell membranes. Haem contains iron that catalyses the generation of reactive oxygen species through the Fenton and Haber-Weiss reactions [8]. To avoid such damage, the acute phase protein haptoglobin (Hp) binds to the free Hb and thus prevents cellular damage by oxidative-stress after haemolysis [9]. These Hp:Hb complexes are phagocytized by macrophages and monocytes, which recognize the complex through their membrane-bound CD163 receptor [9], which might be a reason why hypohaptoglobinaemia (i.e., low detectable Hp) is often observed in malaria endemic areas [10].

The *HP* gene is located on chromosome 16 (location: 16q22.1) and consists of two different loci: haptoglobin alpha (*Hp-α*) and haptoglobin beta (*Hp-β*), coding for the α-chain and β-chain, respectively, of the Hp protein [11]. The locus *Hp-α* can consist of the Hp^1^ allele or the Hp^2^ allele, which results in three different phenotypes: the homozygotes Hp1-1 and Hp2-2 and the heterozygote phenotype Hp2-1 [11]. The Hp2 allele originated from an intragenic duplication initiated by non-homologous crossing-over of two Hp1 alleles [12]. The effect of this intragenic duplication can be seen on protein level after the denaturation of Hp by the two different sizes of the α subunits of the Hp protein, the Hp1α subunit (8.9 kDa) and the Hp2α subunit (16 kDa) [11,12].

The different Hp phenotypes influence the progression of various infectious and inflammatory diseases, including malaria, due to their phenotype-dependent binding affinity to Hb (Hp1-1 > Hp2-1 > Hp2-2) and the CD163 receptor on monocytes and macrophages (Hp2-2 > Hp2-1 > Hp1-1) [11]. During the last decade, the role of Hp phenotypes in malaria has been controversial. Some studies suggested that the Hp2-2 phenotype protects against severe *P. falciparum* infection [13,14], while other epidemiological case control studies did not find such results [15,16].

A fourth phenotype, the Hp0-0 phenotype (i.e., ahaptoglobinaemia), with no detectable Hp levels is found to be abundant in sub-Saharan Africa and has been reported to be present in 46.7% of the population living in Mali [17]. In Africans, ahaptoglobinaemia might be caused by the A-61C single nucleotide polymorphism (SNP), which is found to be associated with the Hp2 allele [18,19]. The A-61C SNP has been suggested to be the cause of the controversy between earlier studies [18]. Furthermore, ahaptoglobinaemia has been shown to be associated with malaria endemicity [20], and hypohaptoglobinaemia as an epidemiological and clinical indicator for malaria [10].

The CD163 receptor is known to induce an anti-inflammatory response via the secretion of IL-10 after the binding of Hp:Hb complexes on circulating monocytes and macrophages [21,22]. In malaria, circulating sCD163 levels have been shown to be higher in children with uncomplicated malaria compared to severe malaria cases and levels of sCD163 were higher in all patient groups compared to healthy individuals, indicating an important role of this mediator during malaria episodes [23]. Previous *in vitro* stimulation assays investigating the response induced by Hp1-1:Hb and Hp2-2:Hb complexes in macrophages showed an increased release of IL-10 and IL-6 upon stimulation with the Hp1-1:Hb complexes compared to the Hp2-2:Hb complexes, suggesting a more anti-inflammatory profile in the former [24].

The aim of this study was to investigate the Hp prevalence in the Fulani and Dogon ethnic groups, which are known to have different susceptibility to malaria. Furthermore, the cytokine profile and soluble mediators linked to these Hp phenotypes were elucidated both *ex vivo* and *in vitro*. This paper has further elucidated some aspects why Fulani individuals are less susceptible to malaria, which might contribute to future individual risk estimation to *P. falciparum* infection.

## Methods

### Study area

Two different cohorts were included in this study and both cohort studies were conducted in the same rural area of the Dogon Valley, located approximately 850 km from the capital of Bamako, Mali. The first cohort comprises individuals recruited in 2008 and the second cohort comprises individuals recruited in 2001. The malaria transmission in the area is meso-endemic with intense transmission during the rainy season that usually extends from June-October. The study area has been described in details elsewhere [2].

### Study population

In the first cohort, 77 children aged between two and ten years belonging to either the Fulani or Dogon ethnic group were recruited. In total there were 40 Dogon children of which 20 were infected and 37 Fulani children of which 14 were infected. In the second cohort, 82 children and adults, aged between 11–68 years were recruited. In total, there were 46 Dogon individuals of which 20 were infected and 36 Fulani individuals of which 5 were infected. Together, there were 159 individuals examined in the two cohorts combined. A thick blood smear was made from each donor and the slides were stained in 3% Giemsa and examined for the presence of *P. falciparum* parasites. Malaria infection was defined as having a positive thick blood smear with or without any malaria symptoms. Axillary temperature was measured in all individuals and symptomatic malaria was defined as fever ≥ 37.5°C, plus the presence of any density of parasites in the blood. All infected individuals in our study had asymptomatic malaria. A shorter description of the study population comprising the individuals enrolled in the two study cohorts can be seen in Table 1. Peripheral blood samples were collected and plasma was separated by centrifugation and stored at −80°C until used in assays. All samples were collected at the end of the rainy season (October-November). Written informed consent was obtained from each patient or the children’s guardians before inclusion in this study. This study was approved by the institutional review boards of the University of Bamako Mali (N°08_64/FMPOS), and by the Swedish research ethical committee (03–536). The study populations from the two cohorts used in this study have been described in details elsewhere [4,6].

**Table 1:** Characteristics of the study population from the two cohorts

	**2001 cohort (n = 82)**		**2008 cohort (n = 77)**	
**Characteristics**	**Fulani**	**Dogon**	**Fulani**	**Dogon**
**Age (range)**	11-68	11-63	2-10	3-9
**Individuals (n)**	36	46	37	40
**Infected (%)**	13.8	43.5	37.8	50.0

### Haptoglobin phenotyping by western blot

Hp phenotypes were determined by a modification of the methodology used by Beutler *et al.*[25]. Briefly, plasma samples were diluted ten times with Tris-Hank’s + 0.02% NaN_3_ and thereafter 10 μl of the diluted plasma samples were added to 10 μl of sample buffer (2.3% sodium dodecyl sulphate, 10% glycerol, 62.5 mM Tris (pH 6.8), 0.2% bromophenol blue and 5% β-mercaptoethanol) and the mixtures were boiled for 5 min. After boiling, 5 μl of sample was loaded on a self-cast 15% polyacrylamide gel (9 × 10 cm). Pooled human Hp (Sigma-Aldrich, St Louis, USA), diluted to 0.1 mg/ml was included as a positive control. Electrophoresis was performed in 1.5 M Tris–HCl (pH 6.8-8.8) for 15 min on 100 V followed by 60 min on 200 V (Bio-Rad; PowerPac, Hercules, CA, USA) and transferred to a nitrocellulose membrane (Bio-Rad; Trans-Blot semi-dry transfer cell). The membranes were blocked in 5% dry milk powder in 0.05% Tris-buffered saline (TBS) (10 mM Tris–HCl (pH 8.0), 150 mM NaCl) for one hour. The membranes were washed two times in TBS + 0.05% Tween followed by once with TBS and were then incubated overnight with a 1:1,000 dilution of a polyclonal rabbit anti-human Hp antibody (Abcam, #ab97976, Cambridge, UK). After washing, the membranes were incubated with a horseradish peroxidase (HRP)-conjugated secondary goat-anti-rabbit IgG antibody (Abcam, #ab97200) in a 1:10,000 dilution. After a final washing step, HRP substrate (Thermo Scientific, Rockford, USA) was added to the membranes and immediately afterwards a photo was taken with Biorad Chemi Doc TM XRS system (Biorad, Hercules, CA, USA).

### Quantification of sCD163 in plasma samples using ELISA

sCD163 concentrations were quantified by a commercially available sandwich ELISA, according to the manufacturer’s instructions (R&D Systems®, Minneapolis, MN, USA). All samples were diluted 1:100 and tested in duplicates. The optical density was measured at 450 nm (Molecular Devices, Vmax Kinetic Microplate Reader, Menlo Park, CA, USA). The concentrations were calculated from a standard curve with eight dilutions of lyophilized recombinant human CD163 protein (range 10.000-78 pg/ml).

### Quantification of cytokine levels in plasma using ELISA

Levels of IL-6, IL-10, TNF and IFN-γ were quantified using ELISA kits with pairs of capture and detection antibodies. The kits were used according to the manufacturer’s recommendation (Mabtech AB, Nacka Strand, Stockholm, Sweden). All samples were diluted 1:2 and tested in duplicates. The enzyme-substrate reaction was developed using p-nitrophenyl-phosphate (Sigma-Aldrich) and the optical densities were measured at 405 nm in an ELISA reader (Molecular Devices, Vmax Kinetic Microplate Reader). The concentrations were calculated from a standard curve obtained from eight dilutions of lyophilized native human IL-6, (range 10,000-3 pg/ml), IL-10, (range 3,160-1 pg/ml), TNF, (range 10,000-3 pg/ml) and IFN-γ recombinant proteins (range 3,160-1 pg/ml). For measuring IFN-γ in the supernatants (generated from the *in vitro* stimulation assays), the samples were run undiluted.

### Haptoglobin:haemoglobin complex formation

For the formation of Hp:Hb complexes, human Hp1-1, Hp2-2 and Hb were all purchased as a lyophilized powder (Sigma-Aldrich). Hb was added in excess (at a 10:1 ratio) to ensure complete Hp saturation to the two Hp proteins (in separate containers) and the mixtures were incubated overnight at 4°C. Of the mixed Hb and Hp suspension, a total sample volume of 2 ml was loaded onto the column and the Hp:Hb complexes were diluted out via fast protein liquid chromatography on a Hiload Superdex 200 pg (16/600) prep grade column (GE Healthcare Life Sciences, Buckinghamshire, UK) that was run for six hours with a flow rate of 0.25 ml/min using PBS. Approximately 10 ml elute was collected in different fractions that were pooled using a 100kD Millipore filter, with a centrifugation speed of 4,000 rcf for 5 min down to 1 ml sample. The concentration of the generated Hp:Hb complexes were quantified by Bradford assay (Bio-rad) and analysed by Nanodrop spectrophotometer ND-1000 (Thermo scientific, Wilmington, DE, USA).

### Isolation and stimulation of peripheral blood mononuclear cells

Venous blood (10 ml) from anonymous blood donors (Skanstull blood bank, Stockholm, Sweden) was obtained in EDTA tubes (BD, Biosciences, Plymouth, UK). The PBMCs were isolated by Ficoll-paque (GE Healthcare Biosciences AB, Uppsala, Sweden) gradient centrifugation. The cells were collected and washed twice in phosphate-buffered saline (PBS) followed by trypan blue exclusion. The cells were diluted to a final concentration of 1 × 10^6^ cells/ml in culture medium (RPMI-1640 (Gibco, Invitrogen, Auckland, New Zealand) supplemented with 1.0% heat inactivated foetal bovine serum (FBS) (Gibco, Invitrogen), 20 mM HEPES, 2 mM L-glutamine, penicillin (100 U/ml) and streptomycin (100 μg/ml) (all from HyClone Laboratories, Inc, South Logan, UT, USA). The cells (3 × 10^5^) were incubated in 48-well plates (Corning, NY, USA) either with the addition of culture medium only or stimulated with Hp1-1:Hb (10 μg) or Hp2-2:Hb (10 μg) at 37°C in 5% CO_2_ for three hours (flow cytometry) or 24 hours (cytokine measurements). After incubation, cell-free supernatants were collected by centrifugation and stored at −80°C until further analysed.

### Flow cytometry

After stimulation, the cells were harvested and washed once with FACS buffer (2 mM EDTA, 0.1% BSA in PBS). The cells were pre-incubated with 10% normal human AB serum for 10 min to block Fc receptors and thereafter stained with titrated amounts of mouse anti-human CD14-FITC (clone: MφP9) (BD Biosciences, Pharmingen) and CD163-PE (clone: GH1/61) (Biolegend). Corresponding isotype-matched antibodies were used as negative controls. Cells were acquired using BD FACSVerse flow cytometry (Becton Dickinson) and analysed with FlowJo software V.10 (TreeStar, Ashland, OR, USA).

### Cytometric bead array

The concentration of cytokines in cell-culture supernatants were measured using the cytometric bead array technique. The human inflammatory cytokine kit (IL-1β, IL-6, IL-8/CXCL8, IL-10, IL-12p70, and TNF) (BD Biosciences, San Diego, CA, USA) was used. The samples were run according to the recommendations by the manufacturer, but with an extended standard curve (a top-standard of 10,000 pg/ml was used instead of 5,000 pg/ml). All samples were tested undiluted. The samples were acquired using a BD FACSCalibur flow cytometer and analysed with FCAPArray v1.0.1 software (SoftFlow, Pécs, Hungary). Calibration of the flow cytometer was performed using BD CaliBRITE Beads and BD FACS-Comp (BD, Biosciences Pharmingen).

### Statistical analysis

Fisher’s exact test was used to compare Hp phenotype prevalence between the ethnic groups. Kruskal Wallis non-parametric test was used to analyse differences in cytokine levels between multiple groups and Mann–Whitney non-parametric test was used to detect differences between two groups. Spearman rank correlation was used to analyse correlations between cytokine levels and age. Multiple logistic regression analysis was performed to investigate Hp phenotypes prevalence in relation to infection status. A multiple linear regression and logistic regression model were used to investigate the relationship between cytokines and inflammatory mediators with Hp phenotypes. Due to high number of samples with undetectable levels, the data was treated as binary (detectable or not) in the multiple logistic regression model, adjusted for age, infection status, Hp phenotypes and ethnicity. In the non-parametric tests the data was treated as actual values. Wilcoxon Signed rank test was used to investigate cytokine profiles and cell data in the *in vitro* stimulation experiments. Significant difference was assumed when P < 0.05. The data was analysed with Statview version 5.0.1 and/or Stata 12.0.

## Results

### Haptoglobin phenotype detection by western blot

**Figure 1:**
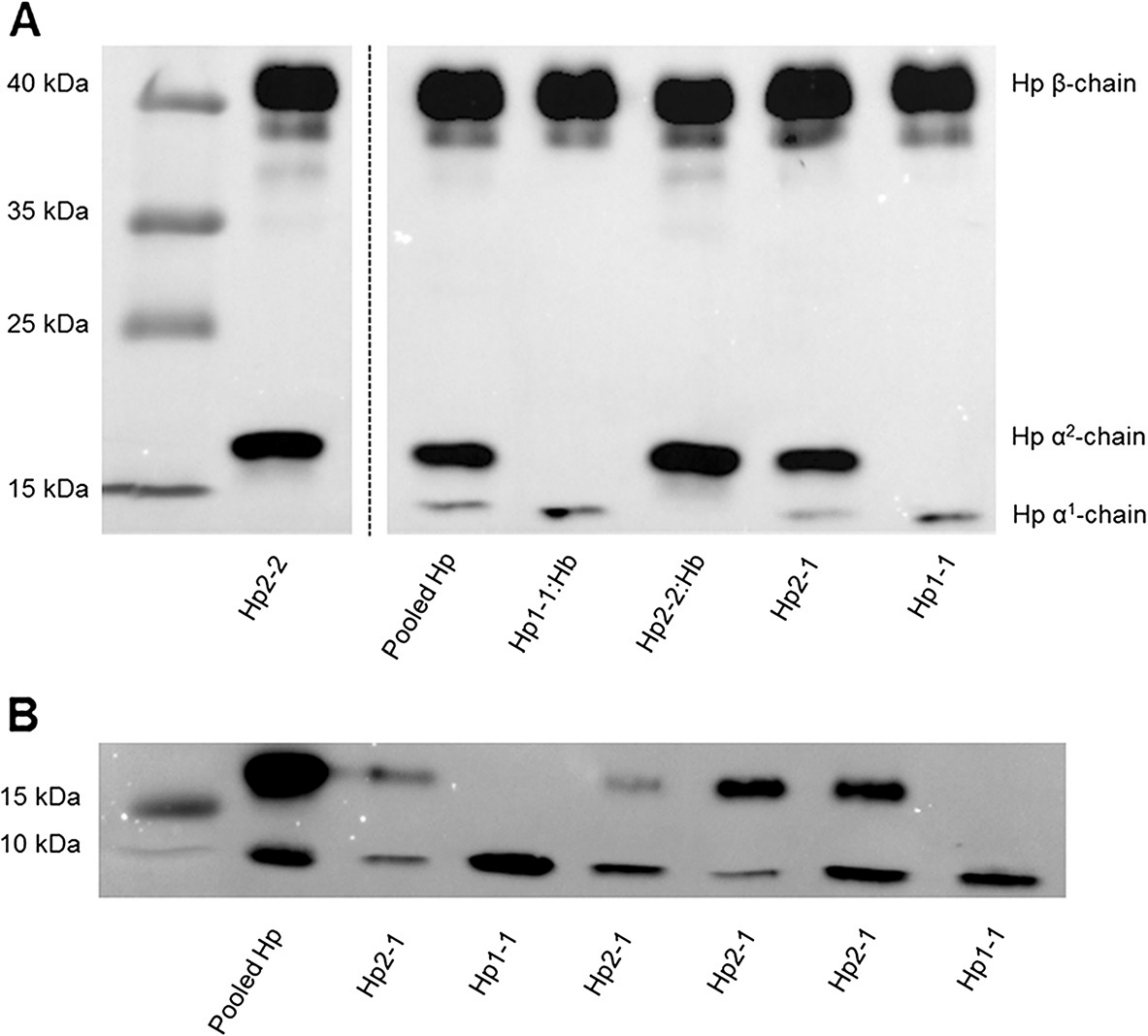
**Determination of haptoglobin phenotypes by Western Blot.** In total 159 plasma samples were analysed for haptoglobin phenotype. Figure **A** shows a membrane with bands correcponding to the alpha-subunits (8.9 kDa/16 kDa) and the betha-subunit (40 kDa) of the haptoglobin protein. The membrane shows bands for the pooled human haptoglobin, the two Hp:Hb complexes (Hp1-1:Hb and Hp2-2:Hb) and plasma samples from the cohort corresponding to the Hp1-1, Hp2-1 and the Hp2-2 phenotype in similar band patterns. One lane was cut out from the gel due to individual variation in the haptoglobin concentrations. Figure **B**, shows a representative membrane of the α-subunits of the haptoglobin protein in different plasma samples as well as the pooled human haptoglobin protein that was included as a positive control on each membrane.

The collected plasma samples were analysed for Hp phenotypes by Western blot (Figure 1). A membrane with the control protein (pooled Hp), the two Hp:Hb complexes (Hp1-1:Hb and Hp2-2:Hb) as well as plasma samples from the cohort corresponding to the Hp1-1, Hp2-1 and the Hp2-2 phenotypes are shown in Figure 1A. The β-chain of the Hp can be seen at 40 kDa, the Hpα^2^-chain at 8.9 kDa and the Hpα^1^-chain at 16 kDa, respectively. Both the alpha and the beta chain were visible in the pooled Hp (used as a positive control), which corresponded to the bands visualized in the plasma samples (Figure 1A). A representative membrane showing the α-units of the Hp protein in different plasma samples as well as the pooled Hp can be seen in Figure 1B. One individual (0.6%) out of all the plasma samples analysed did not give any band pattern after the Western Blot assay was performed, and was thus defined as ahaptoglobinaemic (Hp0-0), and was therefore excluded from further analysis.

### Prevalence of haptoglobin phenotypes in Dogon and Fulani

The Hp phenotype prevalence in Dogon and Fulani individuals can be seen in Table 2. To elucidate if there was a difference in the Hp prevalence between Fulani and Dogon individuals in the total cohort, a Fisher exact test was performed. The results showed that there was a significant difference between Dogon and Fulani individuals (P = 0.017), indicating that the two ethnic groups have different distribution in their Hp phenotypes. To further elucidate which phenotype contributed to this difference, a follow-up test in which individuals with the Hp1-1 *versus* Hp2-1 and Hp2-2 phenotype for corresponding ethnicity were compared. This did not show any significant difference (P = 0.254), indicating that the Hp1-1 phenotype did not contribute to the overall difference seen between Dogon and Fulani. Therefore a comparision between individulas with the Hp2-1 and Hp2-2 phenotype was carried out, in which Fulani individulas were found to have higher prevalence of the Hp2-2 phenotype compared to the Dogon (P = 0.010). The results indicate that the the difference in Hp phenotype distribution between Fulani and Dogon mainly originated from differences within the Hp2-2 phenotype. The observed Hp phenotype prevalence was in Hardy-Weinberg equilibrium (X^2^ = 0.38, df = 1, P = 0.54).

**Table 2:** Prevalence of haptoglobin phenotype in Dogon and Fulani

	**Fulani**		**Dogon**	
	**Prevalence (n, %)**	**Infection rate n (%)**	**Prevalence (n, %)**	**Infection rate n (%)**
**Hp1-1**	32 (44.4)	11 (34.4)	30 (34.9)	10 (33.3)
**Hp2-1**	24 (33.3)	6 (25.0)	47 (54.7)	23 (49.0)
**Hp2-2**	16 (22.2)	2 (12.5)	9 (10.5)	7 (77.8)
**Total**	72 (100)	-	86 (100)	-

### Haptoglobin phenotypes in relation to infection status in Dogon and Fulani

When looking at the Hp prevalence distribution and infection rate in Fulani and Dogon individuals, the results show that within the Fulani, the prevalence of being infected was highest in Hp1-1, intermediate in Hp2-1 and the lowest in Hp2-2 individuals, respectively, while the opposite pattern was observed within the Dogon population, i.e., highest in Hp2-2, intermediate in Hp2-1 and lowest in Hp1-1 individuals (Table 2). To investigate a possible influence of the Hp phenotype on the infection status in Fulani and in Dogon individuals, a logistic regression model was used in which data were adjusted for age. The Hp1-1 phenotype within each ethnic group was used as a reference group in the analysis. The results indicated that the above-mentioned pattern was the same for the odds ratio, which calculates the risk of being infected in Fulani and Dogon individuals with the different Hp phenotypes (Table 3). The regression analysis indicated that within the Dogon, individuals having the Hp2-2 phenotype were more prone to be infected as compared to individuals with the Hp1-1 phenotype (P = 0.041, OR = 6.62 [95% CI: 1.1-40.6]) (Table 3). In contrary, within the Fulani, individuals having the Hp1-1 phenotype might be more prone to be infected compared to individuals having the Hp2-2 phenotype, although this was not statistically significant and should be verified in a bigger sample cohort (P = 0.148, OR = 0.26 [95% CI: 0.05-1.4]) (Table 3).

**Table 3:** Logistic regression analysis of the infection rate on haptoglobin phenotype prevalence in Dogon and Fulani, adjusted for age

	**Odds ratio**		**95% Confidence interval**		**P value**	
**Variable**	**Fulani**	**Dogon**	**Fulani**	**Dogon**	**Fulani**	**Dogon**
Hp1-1	1	1	-	-	-	-
Hp2-1	0.95	2.07	0.3-3.4	0.8-5.5	0.932	0.148
Hp2-2	0.26	6.62	0.05-1.4	1.1-40.6	0.115	0.041

Footnote: Hp1-1 from corresponding ethnic group was used as the reference group.

### sCD163 levels in plasma samples from Dogon and Fulani

**Figure 2:**
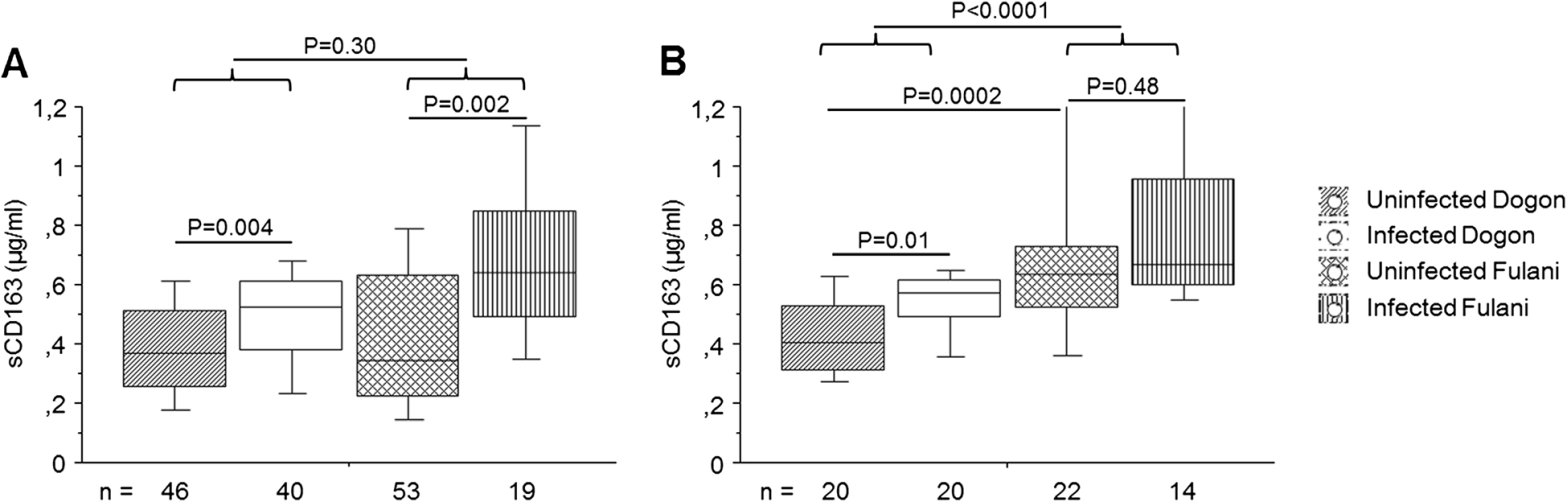
**sCD163 in plasma from Dogon and Fulani individuals.** Blood plasma samples from 158 individuals were analysed for levels of sCD163. Individuals were divided according to ethnicity and infection status. Levels are shown for **A)** the total population and **B)** the children cohort from 2008 (aged two to ten years). The boxes represent the lower quartile (25%) and the upper quartile (75%) with the middle line as the median. The whiskers indicate the 10 and 90% percentiles. Statistical analysis was done with Kruskal-Wallis and Mann–Whitney *U* test.

Plasma samples from Dogon and Fulani individuals were analysed for levels of sCD163. In the total cohort (combining the 2001 and the 2008 cohort), the results showed that there were no differences in sCD163 levels between Fulani and Dogon, irrespective of infection (P = 0.30; Figure 2A). However, when only looking at the children cohort from 2008 aged between two and ten years, Fulani children were found to have significantly higher sCD163 levels compared to Dogon children, irrespective of infection (P < 0.0001; Figure 2B). The results indicate an age-driven difference in sCD163 levels. Interestingly, in the total cohort, the results also showed elevated levels of sCD163 in infected individuals compared to uninfected individuals in both Fulani (P = 0.002) and Dogon (P = 0.004) (Figure 2A), indicating that this anti-inflammatory mediator has an important role during malaria infection. However, when looking at the children cohort the results were different. Infected Dogon children have increased sCD163 levels compared to uninfected individuals of the same ethnicity (P = 0.01), which was not the case between infected and uninfected Fulani children (P = 0.48; Figure 2B). Additionally, uninfected Fulani children showed initially higher levels of this anti-inflammatory mediator, compared to uninfected Dogon children (P = 0.0002; Figure 2B), suggesting that Fulani children have initially higher levels of this mediator during childhood, compared to Dogon. Multiple linear regression analysis of the total cohort (in which data were adjusted for ethnicity, infection status and age) showed the same results: that Fulani had increased levels of sCD163 compared to Dogon (P = 0.028) and that infected individuals had increased levels compared to uninfected individuals, irrespective of ethnicity (P = 0.017).

### Cytokine levels in plasma samples from Dogon and Fulani

**Figure 3:**
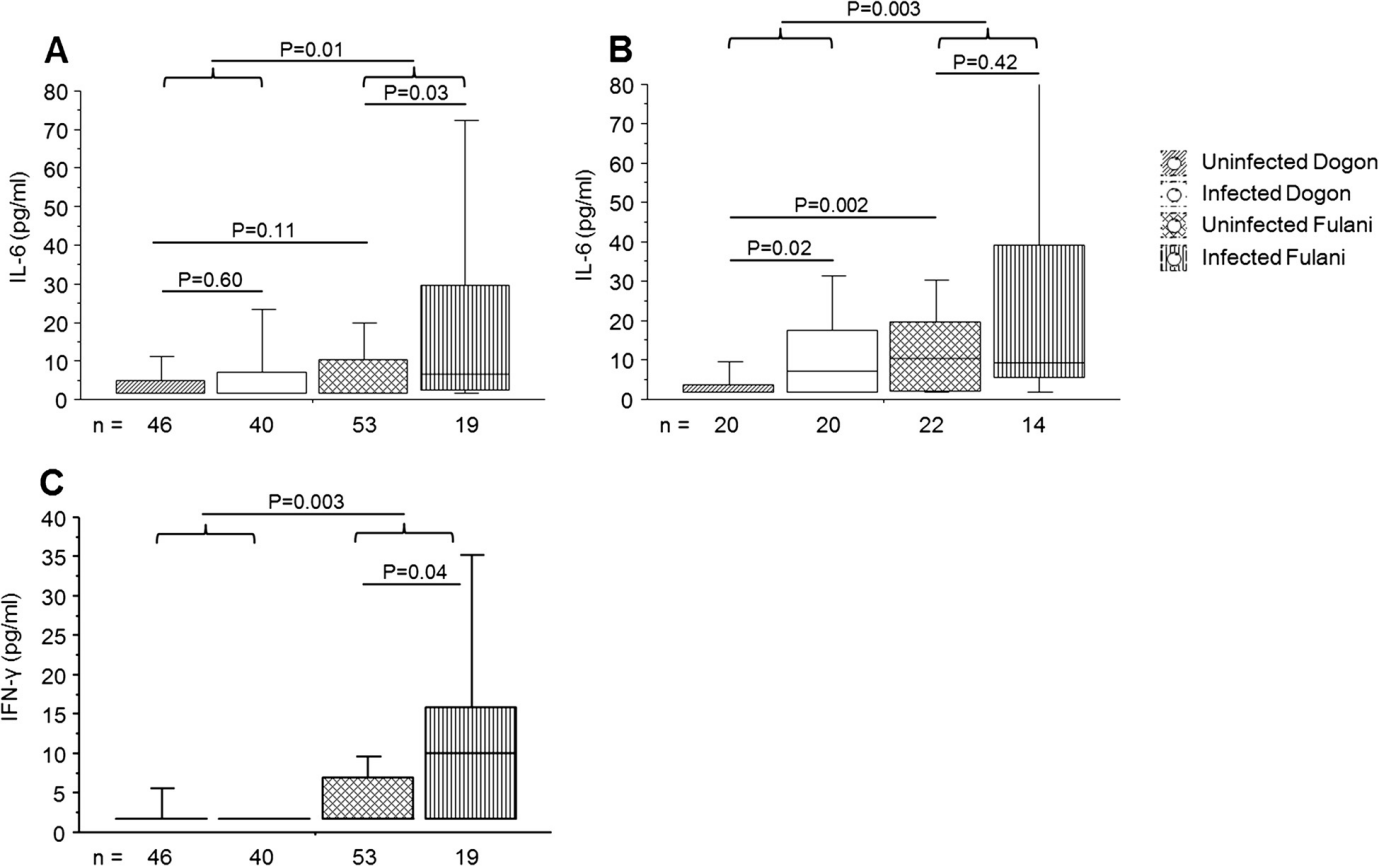
**Cytokine levels in plasma from Dogon and Fulani individuals.** Blood plasma samples from 158 individuals were analysed for levels of IL-6 and IFN-γ. Individuals were divided according to ethnicity and infection status. Levels are shown for **A** and **C)** the total population and **B)** the children cohort from 2008 (aged two to ten years). The boxes represent the lower quartile (25%) and the upper quartile (75%) with the middle line as the median. The whiskers indicate the 10 and 90% percentiles. Statistical analysis was done with Kruskal-Wallis and Mann–Whitney *U* test.

Plasma samples from Dogon and Fulani individuals were analysed for levels of IL-6, IL-10, IFN-γ and TNF. In the total population, the results showed that Fulani had significantly higher levels of IL-6 (P = 0.01; Figure 3A) and IFN-γ (P = 0.003; Figure 3C) compared to Dogon. Increased IFN-γ levels was also found in infected Fulani compared to uninfected Fulani (P = 0.04; Figure 3C), while this was not seen between infected and uninfected Dogon (P = 0.31; Figure 3C). When looking at the children cohort from 2008, Fulani children had significantly higher levels of IL-6 compared to Dogon (P = 0.003; Figure 3B) but this was not seen in the 2001 cohort. The highest levels of IFN-γ was found in the infected Fulani group in both cohorts however, due to great numbers of individuals having undetectable levels of IFN-γ, (60% in Fulani and 90% in Dogon), the results should be carefully interpreted. There were no difference in the levels of TNF (P = 0.65) or IL-10 (P = 0.74) in the total cohort between Dogon and Fulani and due to a high numbers of undetectable levels for these cytokines, further subdivision into the respective cohorts was not done.

### Inflammatory mediators in correlation to age

**Figure 4:**
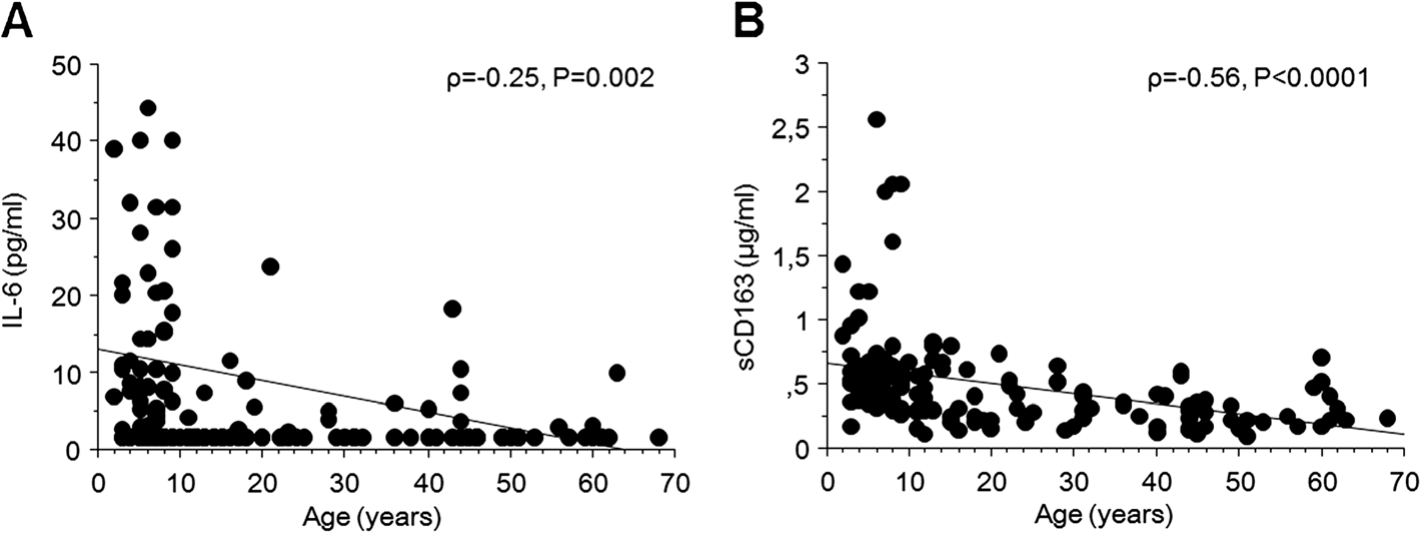
**Correlation between age and inflammatory mediators.** Correlation between age and **A)** IL-6 levels and **B)** sCD163 levels are shown. Statistical analysis was performed with Spearman rank correlation test.

The above-mentioned results suggest that the levels of IL-6 and sCD163 may be age-related. Therefore, a correlation analysis was performed to investigate if these factors correlated to age. A weak negative correlation was found between IL-6 and age (Rho = −0,25; P = 0.002; Figure 4A) and a stronger negative correlation between sCD163 and age (Rho = −0.56; P = <0.0001; Figure 4B). In addition, multiple regression analysis was performed to investigate these mediators in relation to age, adjusted for Hp phenotype, infection status and ethnicity. The results showed an average decrease of 1.6% per year of sCD163 levels (P < 0.001, OR = 0.98 [95% CI: 0.98-0.99]) and also decreased IL-6 levels (P < 0.001, OR = 0.963 [95% CI: 0.94-0.99]).

### Cytokine correlations with haptoglobin phenotypes in Dogon and Fulani

**Figure 5:**
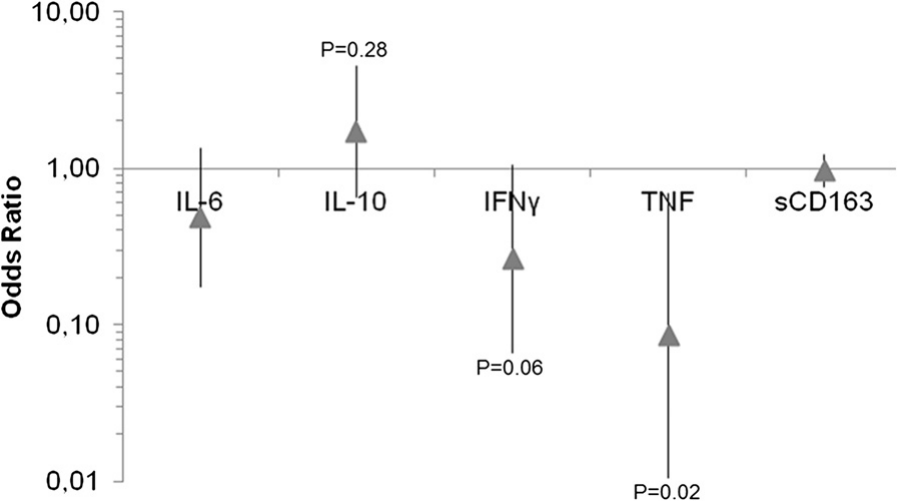
**Odds ratio of inflammatory mediators in relation to the Hp2-2 phenotype.** The odds ratios from the multiple regression analysis for the different cytokines are depicted, adjusted for Hp. The odds ratios are represented for the Hp2-2 phenotype relative to the Hp1-1 phenotype. The arrow represents the odds ratio estimate and the error bars represents 95% CI.

To investigate a possible effect of the Hp phenotypes on the measured cytokine levels the same multiple regression analysis was used, and was adjusted for Hp phenotype, infection status and ethnicity. The results showed that TNF was associated with the Hp1-1 phenotype (P = 0.021, OR = 0.085 [95% CI: 0.01-0.69]), and a similar trend was also seen for IFN-γ (P = 0.06, OR = 0.26 [95% CI: 0.07-1.06]) (Figure 5). When comparing the odds ratios for the total population, almost 12 times higher TNF and four times higher IFN-γ were found for the Hp1-1 phenotype compared to the Hp2-2 phenotype (Figure 5). No other inflammatory mediators were found to be associated with any of the different phenotypes (Figure 5).

### Cytokine profiles from Hp1-1:Hb and Hp2-2:Hb complex stimulated PBMCs

**Figure 6:**
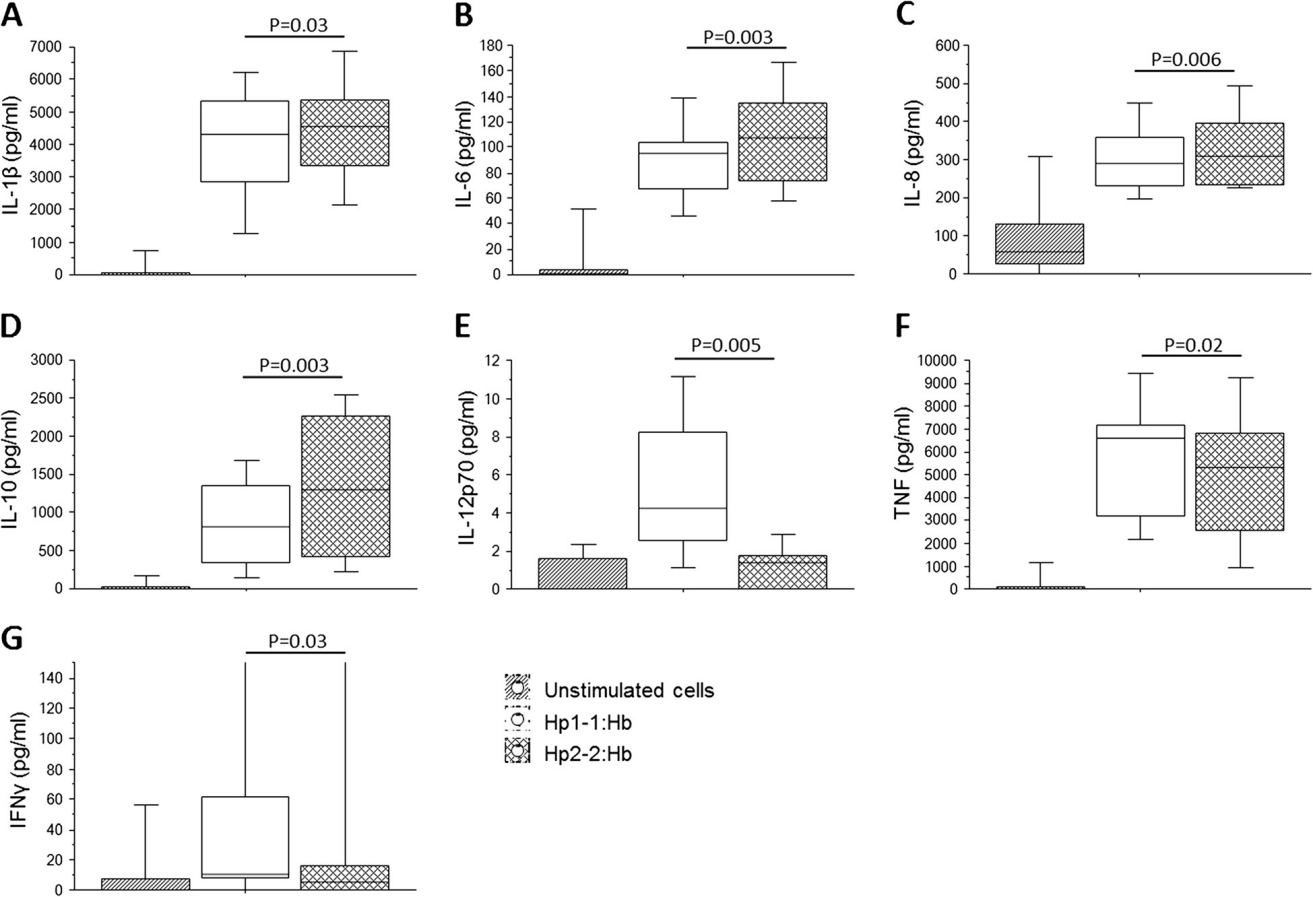
**Cytokine profiles in supernatants from in vitro stimulation assays with Haptoglobin:Haemoglobin complexes.** Isolated PBMCs were stimulated with Hp1-1:Hb or Hp2-2:Hb (both 10 μg/ml) for 24 hours and thereafter corresponding supernatants were collected and analysed for various cytokines. Levels of **A)** IL-1beta, **B)** IL-6, **C)** IL-8, **D)** IL-10, **E)** IL-12p70, **F)** TNF and **G)** IFN-γ from 11 independent experiments are shown. The boxes represent the lower quartile (25%) and the upper quartile (75%) with the middle line as the median. The whiskers indicate the 10 and 90% percentiles. Statistical analysis was done with Wilcoxon Signed ranktest.

To get further information about the different immune responses elicited by the Hp:Hb complexes, an *in vitro* assay was set up in which PBMCs, obtained from Swedish healthy donors, were stimulated with either Hp1-1:Hb or Hp2-2:Hb complexes for three hours (flow cytometry) or 24 hours (cytokine measurements). Thereafter cells were collected and analysed for CD163 expression and the collected supernatants were analysed for different cytokines. The results showed that the CD14^+^ monocyte population had a reduced percentage of cells expressing CD163 (P = 0.002) as well as decreased GeoMFI of the receptor (P = 0.002) when stimulated with the two complexes compared to the unstimulated cells (Additional file 1). Further, the results show that stimulation with the Hp2-2:Hb complexes leads to significantly higher levels of IL-1β (P = 0.03; Figure 6A), IL-6 (P = 0.003; Figure 6B), IL-8 (P = 0.006; Figure 6C) and IL-10 (P = 0.003; Figure 6D) compared to stimulation with Hp1-1:Hb complexes. Stimulation with the Hp1-1:Hb complexes leads to significantly higher levels of IL-12p70 (P = 0.005; Figure 6E), TNF (P = 0.02; Figure 6F) and IFN-γ (P = 0.03; Figure 6G) compared to the Hp2-2:Hb complex.

## Discussion

In this study it was shown that Hp phenotype prevalence can differ between sympatric ethnic groups and that individuals respond differently upon a malaria infection based on their Hp phenotype. The Fulani were shown to have a higher Hp2-2 prevalence compared to the Dogon. This Hp2-2 phenotype was associated with a higher susceptibility to *P. falciparum* infection in the Dogon, but not in the Fulani. Moreover, in concordance with previous studies, the Fulani were shown to have increased circulating inflammatory mediators (IL-6, IFN-γ) and additionally also higher sCD163 levels in their blood, compared to the Dogon. Multiple regression analysis revealed that the Hp1-1 phenotype was associated with higher levels of the pro-inflammatory cytokines; TNF and IFN-γ, as compared to the Hp2-2 phenotype. In addition, *in vitro* assays showed that PBMCs obtained from normal healthy individuals secrete high levels of pro-inflammatory cytokines (IL-12p70, TNF and IFN-γ) in response to Hp1-1:Hb complexes over Hp2-2:Hb, while high levels of both pro- (IL-1β) and anti-inflammatory cytokines (IL-6, IL-10) as well as a chemokine (IL-8) were secreted in response to Hp2-2:Hb complexes over Hp1-1:Hb.

When the Hp prevalence in this study was compared to the Hp prevalence reported in a previous study investigating the Hp phenotype prevalence in different northern and western regions of Africa, including Mali [17], a difference in the Hp phenotype prevalence was seen. In this study, the Hp0-0 prevalence was only 0.6%, while it was reported to be 46.7% in the earlier study. However, it was also shown in the same study that geographical differences exist and that in our study area, the prevalence of Hp0-0 is rare, which could explain the low number of individuals found to be Hp0-0 in this study. Also, the malaria transmission in the study area is not intense and previous studies have shown that the frequency of Hp0-0 is higher in endemic compared to low transmission areas [20]. In addition, the total Hp phenotype prevalence of 15.8% for the Hp2-2 phenotype found in this study (calculated from the Hp2 allele frequency (n = 158, Χ^2^ = 12.16, df = 2, P = 0.002) [17]), was significantly lower than the expected Hp2-2 prevalence of 31.0% reported in Mali. However, this prevalence of 15.8% is in accordance to the Hp2-2 phenotype prevalence of 14% found earlier this year in Kenya [26]. Another explanation for the differences seen between these studies could also be the different methodology used to phenotype the different Hp proteins.

Multiple studies in West Africa have shown that the Hp1-1 type is associated with an increased susceptibility to malaria [13,14] as well as with placental malaria [27]. However, in a study with Ghanaian children, no associations between malaria and Hp phenotypes were found [16]. More recently, genotypic analysis tried to explain this controversy by the Hp2-2 associated A-61C SNP, which might have acted as a confounder in the Ghanaian study [18]. Contrary to these previous studies done in West Africa, Mendonca *et al.* reported an increased risk of developing symptomatic malaria in Brazilian individuals with the Hp2-2 genotype and an opposite association in individuals with asymptomatic malaria [28]. Taking these previous studies into account, a decreased risk of malaria infection in Hp2-2 individuals, (i.e., a higher Hp2-2 prevalence in Fulani who are known to be less susceptible), was thus hypothesized. Indeed, the results showed that Fulani individuals had a higher Hp2-2 prevalence compared to Dogon. In Fulani, the odds ratio of infected *versus* uninfected individuals were decreasing from Hp1-1 > Hp2-1 > Hp2-2, although this was not statistically significant. Intriguingly, in the Dogon individuals, the opposite pattern was observed; the risk of being infected was increasing from Hp1-1 > Hp2-1 > Hp2-2. Thus, ethnicity seems to be an important confounder which might explain the controversy seen between different studies in Africa.

It has been noticed that different Hp have different binding affinity to Hb, one could therefore speculate that the different Hb types could also have different binding affinity towards the different Hp proteins, but this is not yet known. Hb can differ in their distribution and depending on ethnic groups and area, therefore, depending on which Hb type one has one could possibly influence the responses mediated once in complex with the Hp. However, Modiano *et al*. [29] investigated the prevalence of known classical malaria-resistance genes (HbC and HbS among others) in Burkina Faso where people from Fulani live in sympatry with their neighbouring ethnic groups Mossi and Rimaibe, and could not find any difference between the groups for HbS but a higher frequency of HbC in the Mossi and Rimaibe groups, which was in contrary to what was expected.

Fulani are known to have higher malaria-specific antibody titers and to mount a stronger inflammatory response compared to Dogon [3,4,30]. In a previous study within the same children cohort, initially higher levels of IFN-α, IFN-γ, IL-6 and IL-12p70 in Fulani compared to Dogon children were found [3]. The increased IL-6 and IFN-γ levels were also found in this study and in addition, the Fulani children were also shown to have initially higher levels of sCD163, regardless of *P. falciparum* infection. Fulani children, thus, not only have higher pro-inflammatory cytokines, but also have initially higher levels of the anti-inflammatory mediator sCD163. These initially higher levels of both pro- and anti-inflammatory mediators might be one of the possible reasons why Fulani are less susceptible to malaria and seem to cope better with a malaria infection. Additionally, in the total population the results showed elevated sCD163 levels upon *P. falciparum* infection, irrespective of ethnicity. This finding is in accordance with a previous study from Ghana also reporting increased sCD163 levels in asymptomatic malaria cases [23]. Both studies thus indicate an important role of this mediator during malaria. In order to investigate a possible influence of ethnicity, infection and age on the circulating cytokines and mediators, a multiple regression analysis was performed in which the data were corrected for these confounders. The analysis showed that even after such correction, levels of sCD163, IL-6 and IFN-γ were increased in Fulani compared to Dogon.

The naturally acquired immunity against *P. falciparum* is of critical importance as shown by devastating mortality numbers in early European expeditions in Africa and Javanese transmigrate studies [31]. In holo-endemic areas such as in Mali, a naturally acquired immunity reduces clinical episodes in chronically exposed adults as compared to children [32]. The negative correlation between age and both sCD163 and IL-6 levels seen in this study could thus be explained by the fact that people in Africa develop acquired immunity after repeated exposure to the malaria parasites. In children, who have not yet acquired this immunity, the levels of sCD163 and IL-6 levels seem to be high compared to adults. However, upon infection, both of these cytokines increase drastically as being part of the massive inflammatory response seen during malaria episodes [33].

Since there was a difference in the Hp phenotype prevalence between Dogon and Fulani individuals, a multiple regression analysis was performed in order to elucidate if any of the cytokines was correlated to any of the Hp phenotypes. The results revealed that there was an association between the pro-inflammatory cytokines TNF and IFN-γ and the Hp1-1 phenotype. In the same model it was shown that IL-10 was the only cytokine with an odds ratio favouring the Hp2-2 phenotype, although this did not reach statistical significance. This increased pro-inflammatory cytokine profile for the Hp1-1 phenotype is in concordance with the *in vitro* results presented in this study in which stimulation of PBMCs with Hp1-1:Hb complexes lead to increased levels of the pro-inflammatory cytokines: IL-12p70, TNF and IFN-γ, while stimulation with the Hp2-2:Hb complexes lead to increased levels of IL-1β, IL-6, IL-8 and IL-10. This indicates that the Hp1-1:Hb complexes induce a pro-inflammatory response, while the Hp2-2:Hb complexes can induce a more balanced response with both pro-inflammatory as well as anti-inflammatory cytokines. The *in vitro* data presented here are however in contradiction to the paper by Guetta *et al.*, in which high levels of both IL-10 and IL-6 were found after stimulating macrophages with Hp1-1:Hb complexes [24]. However, in their paper, the PBMCs were pre-stimulated with dexamethasone (an anti-inflammatory and immunosuppressive glucocorticoid) for 18 hours before stimulating them with the complexes, which could potentially favour the secretion of anti-inflammatory cytokines over pro-inflammatory ones.

Based on the results presented here, it could be speculated that within the Dogon, it might be beneficial to have the Hp1-1 phenotype, while within the Fulani it could be more favourable to have the Hp2-2 type. The Hp1-1 type was associated with the pro-inflammatory cytokines TNF and IFN-γ both *ex vivo* and *in vitro* which could help Dogon individuals to clear the infection, since they showed initially lower levels of these cytokines in their blood. In the Fulani, who already have initially higher levels of several pro-inflammatory cytokines, the Hp1-1 type might exaggerate the inflammation up to a point to which it is causing more harm than being protective for the host. Therefore, Hp2-2 type individuals within the Fulani might be better off than the Hp1-1 type individuals when exposed to *P. falciparum* parasites. Nevertheless, due to the relative small sample size in this study and relatively weak p-values, caution must be applied when interpreting these results and the relevance of these findings should ideally be validated in a bigger cohort. Further genotypic research with a larger sample size should aim to investigate differences in Hp phenotype between Dogon and Fulani and may give more information regarding the specific Hp alleles and their influence on the ethnicity-dependent susceptibility to malaria. Such studies, could also aim to evaluate different allelic variants and their influence of malaria disease. Furthermore, the *in vitro* data presented here were performed on blood collected from Swedish healthy blood donors. Future *in vitro* studies with PBMCs isolated from Fulani and Dogon individuals should provide more insight into the true effect of Hp phenotypes on the secreted *in vitro* cytokine profile.

## Conclusions

In this study it was shown that Hp phenotype prevalence can differ between sympatric ethnic groups and that they respond differently to a malaria infection based on their Hp phenotype. Thus, ethnicity seems to be an important confounder and future studies should aim to take this into account when designing immunological studies.

## Abbreviations

Hp: Haptoglobin; Hp:Hb: Haptoglobin:Haemoglobin complex; sCD163: Soluble CD163 receptor; PBMCs: Peripheral blood mononuclear cells.

## Competing interests

The authors declare that they have no competing interests.

## Authors’ contributions

SB, MT-B, OP, OD, CA, PG, and BM conceived and designed the study; SB and OP performed the laboratory experiments; AD, OD, BM, and CA performed recruitment of study subjects; SB, OP and J-OP analysed the data and performed the statistical analysis; SB, OP and MT-B wrote/drafted and finalized the manuscript. The final manuscript was read and approved by all authors.

## Supplementary Material

Additional file 1: **CD163 expression on peripheral monocytes after*in vitro*stimulation.** Isolated peripheral blood mononuclear cells were stimulated with medium only or stimulated with Hp1-1:Hb (10 μg) or Hp2-2:Hb (10 μg) for three hours followed by subsequent staining with monoclonal antibodies for CD14 and CD163 expression. The A) percentages of monocytes expressing the CD163 receptor and B) the mean fluorescent intensity (MFI) of the CD163 receptor on the monocytes from 11 independent experiments are shown. The whiskers indicate the 10 and 90% percentiles. Statistical analysis was done with Wilcoxon Signed rank test. Click here for file
