# Supplementary figures and images for: Haptoglobin phenotype prevalence and cytokine profiles during Plasmodium falciparum infection in Dogon and Fulani ethnic groups living in Mali
Source: Malar J. 2013 Nov 25;12:432. doi: 10.1186/1475-2875-12-432 (PMC4225596; doi:10.1186/1475-2875-12-432)

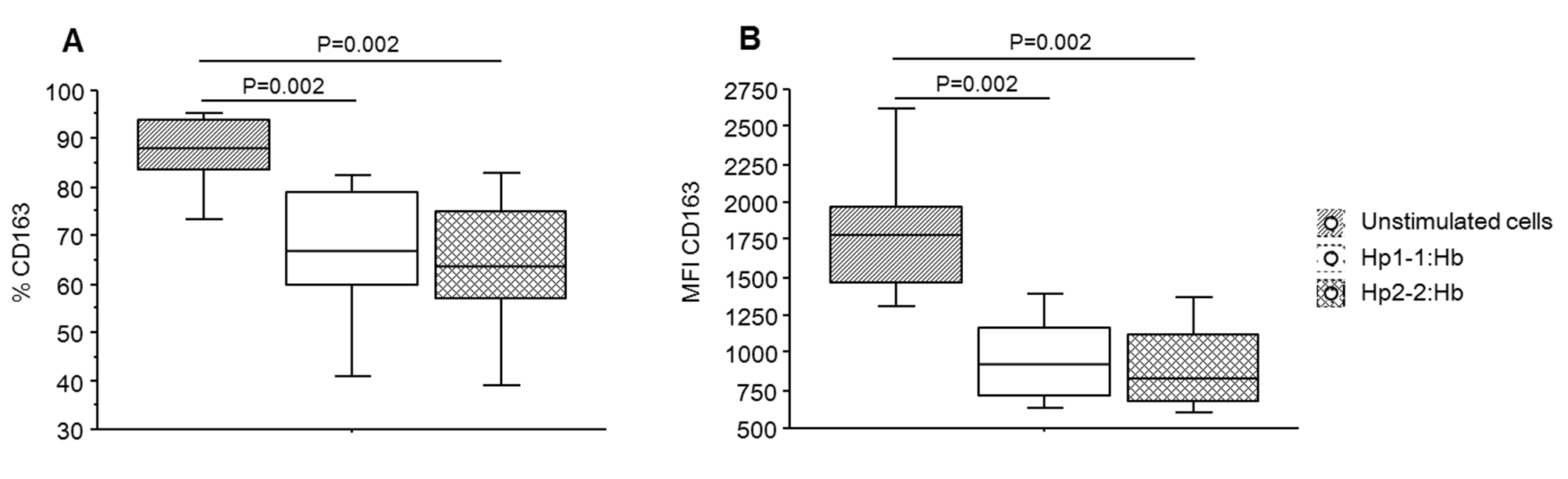

Supplement: Additional file 1: — CD163 expression on peripheral monocytes afterin vitrostimulation. Isolated peripheral blood mononuclear cells were stimulated with medium only or stimulated with Hp1-1:Hb (10 μg) or Hp2-2:Hb (10 μg) for three hours followed by subsequent staining with monoclonal antibodies for CD14 and CD163 expression. The A) percentages of monocytes expressing the CD163 receptor and B) the mean fluorescent intensity (MFI) of the CD163 receptor on the monocytes from 11 independent experiments are shown. The whiskers indicate the 10 and 90% percentiles. Statistical analysis was done with Wilcoxon Signed rank test. [file 1475-2875-12-432-S1.tiff]
